# Prognostic benefit of conversion surgery for HER2 positive stage IV gastric cancer; a case series study of eleven patients treated with trastuzumab-based chemotherapy

**DOI:** 10.1186/s40792-020-00984-w

**Published:** 2020-09-24

**Authors:** Koichi Hayano, Hiroki Watanabe, Takahiro Ryuzaki, Naoto Sawada, Gaku Ohira, Masayuki Kano, Masaya Uesato, Hisahiro Matsubara

**Affiliations:** Department of Frontier Surgery, Chiba University Graduate School of Medicine, 1-8-1 Inohana, Chuo-ku, Chiba, 260-8677 Japan

**Keywords:** Gastric cancer, Human epidermal growth factor receptor-2, Conversion surgery, Trastuzumab

## Abstract

**Background:**

Since the ToGA trial, trastuzumab-based chemotherapy is the standard treatment for HER2 positive stage IV gastric cancer. However, it is not yet clear whether surgical resection after trastuzumab-based chemotherapy (conversion surgery) can improve survival of HER2 positive stage IV gastric cancer. The purpose of this study is to evaluate the prognostic benefit of conversion surgery in HER2 positive stage IV gastric cancer patients.

**Case presentation:**

We retrospectively investigated the medical records of the patients with HER2 positive (IHC3(+) or IHC2(+)/FISH(+)) stage IV gastric cancer treated with trastuzumab-based chemotherapy as the first line treatment. Overall survival (OS) was compared between patients with conversion surgery and without. Eleven HER2 positive stage IV gastric cancer patients treated with trastuzumab-based chemotherapy as the first line treatment were evaluated. Response rate was 63.6%, and 6 of 11 patients could receive conversion surgery. R0 resection was achieved in four patients. In Kaplan–Meier analysis, patients who received conversion surgery showed significantly better OS than those without surgery (3-year survival rate, 66.7% vs. 20%, *P* = 0.03). The median OS of patients who achieved R0 resection is 51.8 months.

**Conclusions:**

Conversion surgery might have a survival benefit for HER2 positive stage IV gastric cancer patients. If curative surgery is technically possible, conversion surgery could be a treatment option for HER2 positive stage IV gastric cancer.

## Background

Gastric cancer is the third leading cause of cancer deaths, and the fifth common cancer globally [1]. The current standard treatment strategy for stage IV gastric cancer is systemic chemotherapy. Since the Trastuzumab for Gastric Cancer (ToGA) trial demonstrated the efficacy and safety of trastuzumab for human epidermal growth factor receptor-2 (HER2) positive gastric cancer [2], trastuzumab-based chemotherapy has been recommended for patients whose tumors had high levels of HER2 protein. It was reported that median overall survival (OS) was 13.8 months in the trastuzumab plus chemotherapy group, compared to 11.8 months in the chemotherapy alone group [2].

Trastuzumab-based chemotherapy occasionally converted an initially unresectable stage IV gastric cancer to a resectable one, and some of those patients actually achieved curative resection. There are several case reports on conversion surgery of HER 2 positive stage IV gastric cancer [3–7], but these reports did not assess long-term survival after surgery. Besides, no cohort study has ever been published on this subject due to the limited population of HER2 positive stage IV gastric cancer (amplification or overexpression of HER 2 is observed in only 7–34% of gastric cancer [8–10]. Therefore, it is still unclear whether conversion surgery has a prognostic benefit for HER 2 positive stage IV gastric cancer. In this study, we investigated prognostic benefit of conversion surgery after trastuzumab-based chemotherapy in HER 2 positive stage IV gastric cancer patients.

## Case presentation

### Patient population

This retrospective study was performed according to the guidelines of the protocols for clinical research approved by the ethics committee at Chiba University Graduate School of Medicine (IRB number 3594). Written informed consent for participation was not required because of the retrospective nature of this study. We retrospectively investigated the medical records of the patients with HER2 positive (immunohistochemistry (IHC) 3+ or IHC2+/fluorescence in-situ hybridization (FISH) positive) stage IV gastric cancer who were treated with trastuzumab-based chemotherapy as the first line treatment from October 2011 to April 2017.

### Treatment and follow-up

The treatment schedule and the dose modification schema of trastuzumab-based chemotherapy have been detailed previously [2]. This chemotherapy was given every 3 weeks. Capecitabine 1000 mg/m^2^ or S-1 80 mg/m^2^ was given orally twice a day for 14 days followed by a 1-week rest. On day 1, Cisplatin 80 mg/m^2^ was intravenously administered. Trastuzumab was also intravenously given at a dose of 8 mg/kg on day 1 of the first cycle, followed by 6 mg/kg every 3 weeks. This combination therapy was continued until disease progression, unacceptable toxicity, or patient refusal. Tumor responses were assessed by CT every 2–4 cycles of the chemotherapy. The tumor markers including carcinoembryonic antigen and CA 19-9, were measured every month. If a patient achieved complete response (CR), partial response (PR), or stable disease (SD) without the appearance of new metastatic lesions, and was predicted to achieve curative surgery, conversion surgery was offered. And then conversion surgery was performed in patients who accepted this surgery. At the beginning of the surgery, intraoperative peritoneal washing and careful observation of peritoneal cavity were conducted. If a patient was diagnosed as CY1 or P1, conversion surgery was not performed. Basically, adjuvant chemotherapy with S-1 was administered after conversion surgery. After these therapies, any additional treatment occurred at the discretion of the treating physician, but basically followed Japanese gastric cancer treatment guidelines [11].

### Statistical analysis

Statistical analyses were carried out using the JMP 13.0 (SAS Institute, Inc., Cary, NC, USA), and for all comparisons, *P* < 0.05 was considered to indicate a statistically significant difference. Kaplan–Meier analysis was performed for OS analysis, and the log-rank test was employed.

## Results

### Patient characteristics

**Fig. 1:**
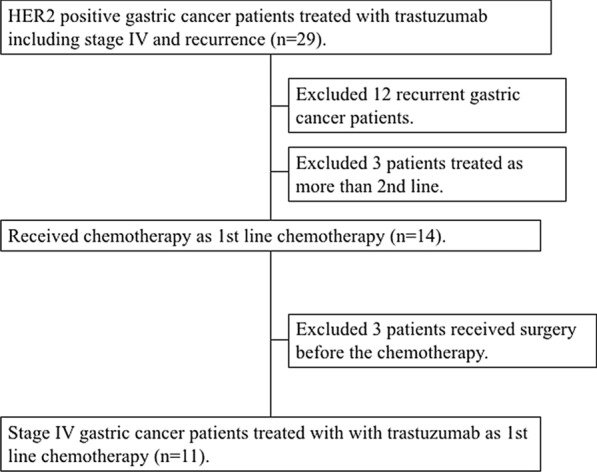
Consort diagram of patients included in this study

We identified 29 consecutive gastric cancer patients (including stage IV and recurrent gastric cancer) who were treated with trastuzumab. 12 recurrent gastric cancer patients were excluded, and 3 stage IV patients were also excluded, because they received trastuzumab as more than 2nd line chemotherapy. Then, 14 patients received the trastuzumab-based chemotherapy as the first line treatment for stage IV gastric cancer, but 3 patients received surgical resection of the main tumor to prevent bleeding, and therefore, a total of eleven patients were eligible in this study (Fig. 1). These subjects included eight men and three women, with a median age of 64.0 years (range 43–78 years). The median follow-up time was 31.5 months. Patients’ characteristics were summarized in Table 1.

**Table 1:** Patient characteristics

Patient demographics	Variables	Value
Sex	Male/female	8/3
Age	Median/range	64.0/43–78
Tumor depth	cT4/T3/T2	6/4/1
Nodal stage	cN3/N2/N1	5/5/1
Hepatic metastasis	Positive/negative	6/5
Distant metastasis (except liver)	Positive/negative	5/6
Peritoneal metastasis	P1/P0 and CY1/P0	2/3/6
Regimen	XPT/SPT	9/2

*P1* peritoneal metastasis, *CY* peritoneal cytology, *XPT* xeloda + cisplatin + trastuzumab, *SPT* S-1 + cisplatin + trastuzumab

### Treatment response and survival after trastuzumab-based chemotherapy

**Fig. 2:**
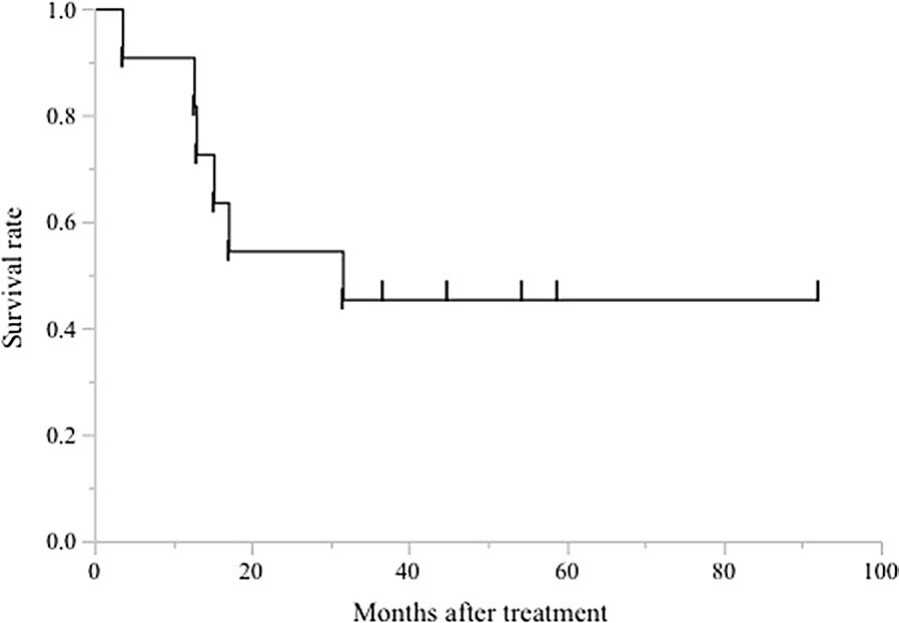
Survival curve for overall survival of 11 patients was analyzed with the use of Kaplan–Meier analysis

Those 11 patients were evaluable for response according to RECIST 1.1. Seven were diagnosed as partial response (PR), one was stable disease (SD) at their best response, and 2 showed progressive disease (PD). Response rate was 63.6%, and the median OS, since the start of trastuzumab-based chemotherapy of those 11 patients was 31.5 months (Fig. 2).

### Comparison between conversion surgery group and chemotherapy alone group

**Fig. 3:**
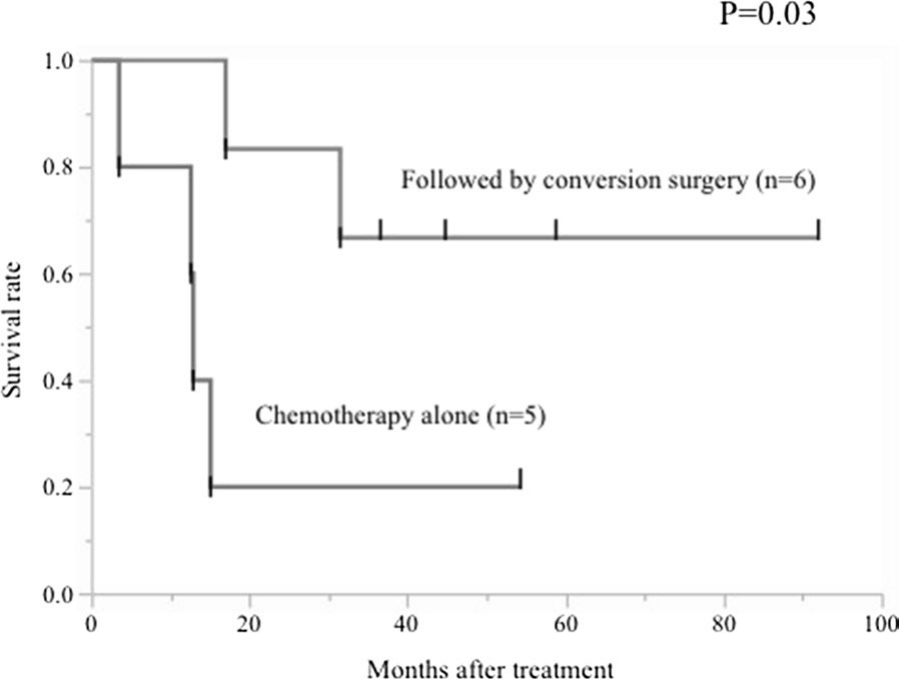
Kaplan–Meier analysis demonstrated that patients who received conversion surgery showed significantly better overall survival than those without conversion surgery (chemotherapy alone) (*P* = 0.03)

Six of eleven patients (PR, 5 cases; SD, 1 case) received surgery after the chemotherapy. The median number of chemotherapy cycles until surgery was 5 (4–14). Total gastrectomy was performed in 4 cases, and distal gastrectomy in 2 cases, and all of them received D2 lymph node dissection. Two cases received hepatectomy with curative intent. R0 resection was achieved in 4 patients, and one patient achieved pathological complete response. Comparison of other characteristics between patients received surgery and those received chemotherapy alone was shown in Table 2. According to Table 2, there was a tendency that conversion surgery was achieved in patients who had no extrahepatic distant metastasis or peritoneal metastases (except only peritoneal cytology positive cases). In addition, no conversion surgery cases had more than or equal to three stage IV factors. Kaplan–Meier analysis demonstrated that patients who received conversion surgery showed significantly better OS than those without conversion surgery (Fig. 3; 3-year survival rate, 66.7% vs. 20%, *P* = 0.03). The median OS of patients who achieved R0 resection is 51.8 months (range 36.6–92.0, all patients are still alive), while two patients with R1 resection died within 3 years since the start of treatment (17 months and 31.5 months).

**Table 2:** Patient characteristics of conversion surgery group and chemotherapy alone group

Patient demographics	Variables	Conversion surgery	Chemotherapy alone
Sex	Male/female	5/1	3/2
Age	Median/range	65.0/61–68	64.0/43–78
Liver metastasis	Positive/negative	3/3	3/2
Distant metastasis (except liver)	Positive/negative	0/6	4/1
Lymph node metastasis	Regional/not regional	0/6	3/3
Peritoneal metastasis	P1/P0 and CY1/P0	1/2/3	2/0/3
Number of stage IV factor	Less than 2/3 factors	6/0	3/2

*P1* peritoneal metastasis, *CY* peritoneal cytology

### HER2 status of tumor after the chemotherapy in conversion surgery cases

Because one patient achieved pathological complete response (no residual cancer cell), surgical specimens of five patients were investigated to evaluate HER 2 status of gastric cancer after the trastuzumab-based chemotherapy. Interestingly, HER2 expression of the primary tumor was found to be negative in all five gastric cancer patients who received conversion surgery.

## Discussion

Standard treatment for stage IV gastric cancer is basically chemotherapy. For HER2 positive stage IV gastric cancer patients, recommended treatment is trastuzumab-based chemotherapy [2]. According to the ToGA trial, the median OS was observed to extend to 13.8 months following trastuzumab therapy [2]. On the other hand, recent studies have shown the prognostic benefit of conversion surgery after chemotherapy in patients with unresectable gastric cancer [12–19]. Especially, some reports suggested prognostic importance of R0 resection after palliative chemotherapy in conversion surgery of stage IV gastric cancer patients who responded to the chemotherapy [15–19]. Therefore, we may recommend conversion surgery after chemotherapy of stage IV gastric cancer, if curative surgery is technically possible. However, regarding conversion surgery for HER2 positive stage IV gastric cancer, only a few case reports were published [3–7], and they did not assess prognostic importance of conversion surgery. Besides, no cohort study has ever been published on this subject because of the limited population of HER2 positive stage IV gastric cancer [2].

In this study, consecutive 11 HER2 positive stage IV gastric cancer patients treated with trastuzumab-based chemotherapy as the first line treatment were retrospectively investigated. Our study demonstrated a good treatment response of trastuzumab-based chemotherapy with 63.6% of response rate, and favorable OS of the conversion surgery group, while the OS of the chemotherapy alone group was almost the same as that of the ToGA trial (12.9 months vs. 13.8 months). Interestingly, the 3-year survival rate of patients who could achieve R0 resection was 100%, which is extremely high compared to that of stage IV gastric cancer patients treated with chemotherapy [20]. Compared to the ToGA trial, the OS of our patients was obviously better (31.5 months vs. 13.8 months). It might be because patients in the ToGA trial did not receive surgery after the chemotherapy, even though some of them might have a chance to receive R0 resection, because resection is not generally recommended for stage IV gastric cancer. Therefore, conversion surgery can be an effective treatment option for HER2 positive stage IV gastric cancer patients. However, our study just showed the prognosis of the conversion surgery group (all of them were treatment responders) and that of the chemotherapy alone group (half of them were non-responders). Therefore, even though we compared them, we could not demonstrate a true survival benefit of conversion surgery. However, the 3-year survival rate of patients who could achieve R0 resection was 100.0% (median OS, 51.8 months), which is extremely high compared to that of stage IV gastric cancer patients treated with chemotherapy [20]. Besides, a paper reported that the median OS of responders in advanced gastric cancer patients treated with trastuzumab was about 20 months [21], which is less than that of the conversion surgery group in our study. Therefore, we believe that our results indirectly suggest prognostic benefit of conversion surgery in HER2 positive stage IV gastric cancer, and we might say that our results were consistent with favorable results on conversion surgery for stage IV gastric cancer patients [12–19]. The indication for conversion surgery is still under discussion. Yoshida et al. proposed a new classification of stage IV gastric cancer according to the biological characteristics [15]. Their classification included four categories as following; category 1 is potentially resectable, category 2 is defined as gastric cancer with a marginally resectable metastasis, category 3 is gastric cancer with a potentially unresectable metastasis of peritoneal dissemination, and category 4 includes gastric cancer with non-curable metastasis. And they suggested that the indications for conversion therapy might include the patients from category 2, some patients from category 3 and a very small number of patients from category 4. In fact, our conversion surgery group included two “category 1” patients, three “category 2” patients, and one “category 4” patient. Thus, the addition of conversion surgery after chemotherapy may result in long term survival in selected patients, but it still remains unclear how is the indications and timing of the operation after the palliative chemotherapy, which should be studied in further investigation.

Besides, we demonstrated that HER2 expression of the primary tumor that was positive before the treatment became negative in all gastric cancer patients who received conversion surgery after trastuzumab-based chemotherapy. Miyake et al. reported that about 11 of 16 HER2 positive gastric cancer patients (about 70%) before first line chemotherapy lost HER2 expression after trastuzumab-based chemotherapy [22]. Moreover, regardless of HER2 expression after first line chemotherapy using trastuzumab, it was reported that continuous use of trastuzumab beyond progression failed to improve survival in a meta-analysis [23]. Therefore, it might be better not to use trastuzumab in adjuvant chemotherapy after conversion surgery, but further investigation will be needed.

Our study has limitations as follows. First, this study is based on single-center data, and the sample size is very small, even though the incidence of HER2-positive gastric cancer is relatively low. Second, retrospective nature is also a limitation of this study. The change of treatment strategy such as surgical technique and chemotherapy during a relatively long study period (7 years) may affect the results. Prospective multicenter study with larger patient population will be needed to confirm our findings in the future studies.

## Conclusions

Though this is a retrospective study with a small sample size, it was demonstrated that trastuzumab-based chemotherapy was effective, and conversion surgery might have a survival benefit for HER2 positive stage IV gastric cancer patients. If curative surgery is technically possible, conversion surgery could be a treatment option for HER2 positive stage IV gastric cancer. Even though diagnosed as stage IV, if HER2 status is positive, patients can have a hope for long survival with trastuzumab-based chemotherapy plus conversion surgery.

## Data Availability

The datasets analyzed during the current study are available from the corresponding author on reasonable request.

## Abbreviations

FISH: Fluorescence in-situ hybridization
HER2: Human epidermal growth factor receptor-2
OS: Overall survival
ToGA: Trastuzumab for gastric cancer
